# Three-dimensional static-fluid MR urography with gradient- and spin-echo (GRASE) at 3.0T: comparison of image quality and diagnostic performance with respiratory-triggered fast spin-echo (FSE)

**DOI:** 10.1007/s00261-022-03418-3

**Published:** 2022-03-02

**Authors:** Wei Wang, Junzhe Yang, Jing Liu, Wei Li, Kai Zhao, Ke Xue, Yongming Dai, Jianxing Qiu

**Affiliations:** 1 Department of Radiology, Peking University First Hospital, No.8, Xishiku Street, Xicheng District, Beijing, 100034 China; 2 MR Collaboration, Central Research Institute, United Imaging Healthcare, Shanghai, China

**Keywords:** GRASE, FSE, 3D, Static-fluid MRU, Urinary tract dilation

## Abstract

**Purpose:**

To compare the performance of 3D MRU based on a breath-hold gradient- and spin-echo (BH-GRASE) technique with conventional 3D respiratory-triggered FSE (RT-FSE) sequence in patients with urinary tract dilation.

**Methods:**

We prospectively included 90 patients with urinary tract dilation who underwent both 3D BH-GRASE and RT-FSE MRU at 3T. The acquisition time of two MRU sequences was recorded. Three readers blinded to the protocols reviewed the image quality using a five-point scale and assessed the diagnostic performance related to urinary tract dilation. The relative contrast ratio (CR) between the urinary tract and adjacent area was measured quantitatively.

**Results:**

Acquisition time was 14.8 s for BH-GRASE MRU and 213.6 ± 52.2 s for RT-FSE MRU. The qualitative image analysis demonstrated significant equivalence between the two MRU protocols. 3D BH-GRASE MRU better depicted bilateral renal calyces than RT-FSE MRU (*p* < 0.05). The CR values of the urinary tract were lower on BH-GRASE MRU compared with RT-FSE MRU (*p* < 0.05). There were excellent agreements in the assessment of urinary tract dilation between BH-GRASE and RT-FSE MRU, including the dilated degree, obstructive level, and obstructive imaging features (inter-sequence *κ* = 0.924–1).

**Conclusion:**

3D BH-GRASE MRU significantly decreased the acquisition time and achieved comparable image quality, urinary tract visualization, and diagnostic performance with conventional 3D RT-FSE MRU. Breath-hold 3D MRU with GRASE may provide a feasible evaluation of urinary tract dilation.

**Graphical abstract:**

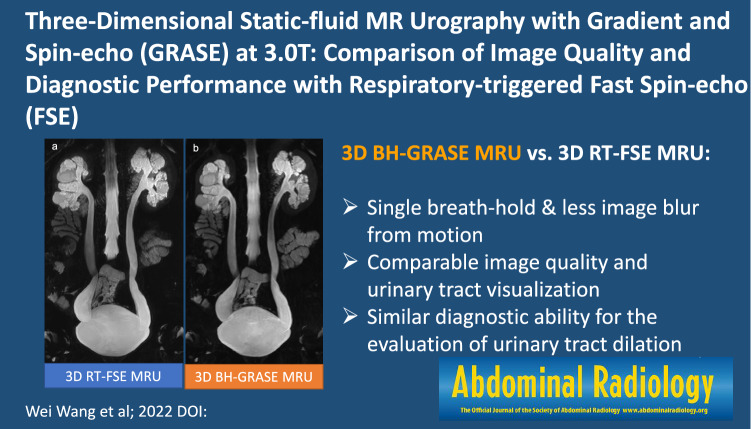

## Introduction

A comprehensive “one-stop-shop” of multi-parametric magnetic resonance urography (MRU) protocol should include basic sequences covering the upper collecting system to the bladder, and other complement sequences such as a heavily T2-weighted cholangiopancreatography (MRCP)-like imaging [1–3]. The latter, which is also called static-fluid MRU, is clinically useful for quickly and directly identifying the urinary tract dilation and obstructive level [3]. The imaging features of the obstructive site on MRU images could help to define the etiology of the urinary tract dilation. Benign obstruction typically smoothly tapers, whereas malignant obstruction may act as an abrupt change in caliber [1]. Compared with conventional excretory MRU or CTU, static-fluid MRU does not depend on the excretion of contrast medium, and it is suitable for patients with poorly excreting, dilated urinary tract [4].

The T2WI MRU can be routinely obtained in two-dimensional (2D) with the breath-hold thick-slab single-shot fast spin-echo or similar thin-section techniques (e.g., half-Fourier rapid acquisition with relaxation enhancement, single-shot fast spin-echo) [4, 5]. Three-dimensional (3D) thin-slice MRU acquisitions could obtain contiguous thin-slice images that allow various post-processing reconstructions in any projection, which may provide better anatomic depiction and lesion conspicuity compared with 2D sequences [6–8]. A conventional 3D MRU uses a respiratory-triggered T2-weighted FSE (RT-FSE) sequence. The drawback is the longer acquisition times over 2D and breath-hold 3D sequences [7]. A higher frequency of blurring artifacts will be seen with respiratory variability caused by a longer acquisition time [9, 10]. Therefore, acceleration of 3D MRU acquisition may decrease the motion-related artifacts.

**Fig. 1:**
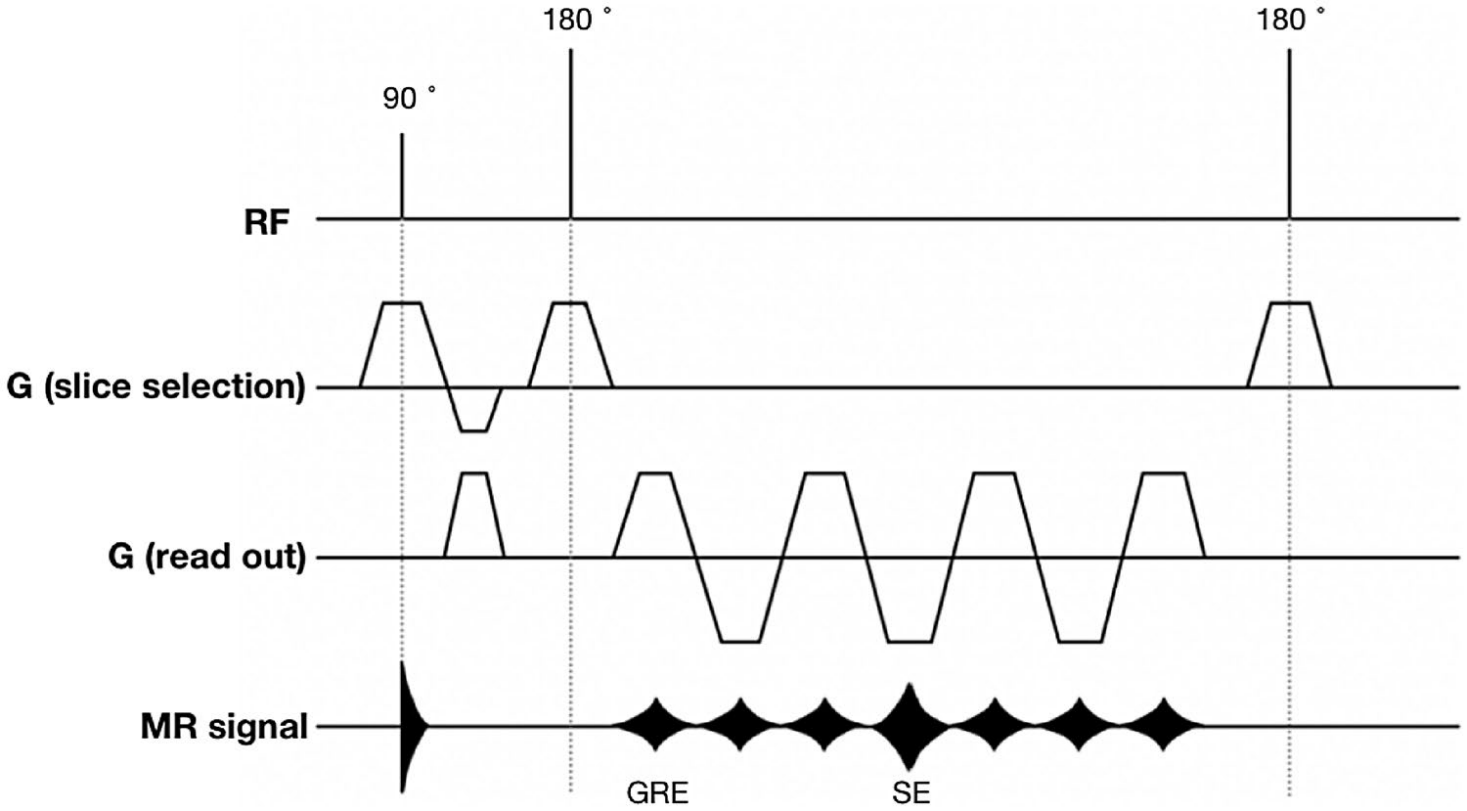
A simplified sequence diagram of the GRASE, including the RF pulse, the slice selection gradient, read-out gradient, and MR signal channels. Between each of 180° RF pulses, several gradient echoes are interleaved (seven in the study). There are additional gradient echoes for readout resulting in shorter acquisition time compared with the FSE sequence. *RF* radiofrequency, *SE* spin-echo, *GRE* gradient-echo

The gradient- and spin-echo (GRASE) sequence is a fast-imaging technique that accelerates acquisition time by combining the gradient- and spin-echo [11], Fig. 1. As several gradient echoes are added into per repetition time interval, the total number of signals per repetition time interval is the product of radiofrequency refocusing pulses and EPI factor [12]. The T2 decay in GRASE is more efficiently used than the spin-echo sequence. GRASE with a shorter acquisition time may contribute to improving the 3D MRU protocol. A few studies have proposed that the breath-hold GRASE (BH-GRASE) technique could significantly decrease the acquisition time in one breath-hold without suffering the image quality, compared with RT-FSE [12–15]. There have been studies reporting the quick sequences for MRU, but only acquired the 2D images [16, 17]. The feasibility of the short-time GRASE in the acquisition of 3D MRU is unclear. Therefore, the purpose of this prospective study was to evaluate the feasibility and performance of BH-GRASE in 3D MRU, by comparing it with conventional 3D RT-FSE MRU.

## Methods

### Patients

This prospective, single-institution study was approved by the ethics committee of our hospital. Written informed consent was obtained from all patients. We continuously recruited patients referred to our MR department for diagnosing, evaluating, or monitoring urinary tract dilation by MRU from January to May 2021. Inclusion criteria were as follows: (1) no contraindications for MRI examination, (2) completion of the two types of MRU techniques described below, (3) no history of urinary tract surgery, (4) visible urinary tract dilation.

### Magnetic resonance imaging sequences and parameters

All MRU examinations were performed on a 3T MR scanner (uMR 790, United Imaging Healthcare, Shanghai, China) using two pieces of 12-channel body matrix coil combined with a 32-channel spine matrix coil. Patients were asked to fast for 6 h and hold urine for 1 h to distend the bladder before the examination. Before the MR examination, routine respiratory training was conducted for every patient including regular breathing and breath-holding.

Imaging protocols included axial T1-weighted sequence, axial and coronal T2-weighted sequences, and axial diffusion-weighted imaging sequence. The 3D MRU sequences were acquired in the coronal plane and have two protocols: (1) BH-GRASE and (2) RT-FSE. The order of the two 3D MRU protocols occurred in a random order, covering the same volume.

3D BH-GRASE MRU was obtained using the GRASE sequence. Acquisition parameters were as follows: repetition time (TR),1300 ms; echo time (TE), 267 ms; flip angle (FA), 180°; parallel acquisition factor, 3; FSE factor, 35; EPI factor, 7; field of view (FOV), 400 × 400 mm; matrix, 213 × 304; resolution (reconstruction) 1.88 × 1.32 × 3 (1.25 × 0.88 × 1.5) mm; slice number, 28; acquisition time, 14.8 s.

Conventional 3D RT-FSE MRU was performed with a FSE sequence. TR/TE, variable depending on respiratory/697 ms; FA, 110°; parallel acquisition factor, 2.7; FSE factor, 205; FOV, 400 × 400 mm; matrix, 285 × 352; resolution (reconstruction) 1.42 × 1.14 × 2 (0.95 × 0.76 × 1) mm; slice number, 42; acquisition time, variable (range 132–392 s).

If the patients could not cooperate with the breath instruction well, we did not repeat the sequence. Both were reconstructed using the maximum intensity projection (MIP) algorithm on the satellite console of the MR unit. The actual acquisition time of both 3D MRU protocols was recorded.

### Image analysis

**Fig. 2:**
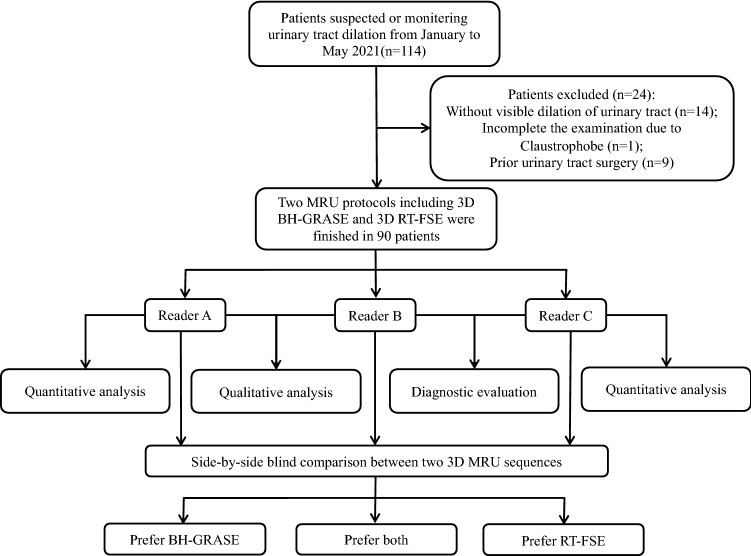
The flowchart of the study. *BH-GRASE* breath-hold gradient- and spin-echo, *RT-FSE* respiratory-triggered fast spin-echo

Three radiologists [Reader *A* (**), *B* (**), and *C* (**), with 9, 11, and 14 years of clinical experience in genitourinary MR imaging, respectively] independently reviewed the FSE MRU and GRASE MRU images on the picture archiving and communication system (PACS) with a 2-week interval to minimize recall bias. The source and MIP images were anonymized and arranged randomly without information on acquisition methods. The readers were free to adjust the window settings according to the reader's experience. The flowchart of the study is demonstrated in Fig. 2.

### Qualitative image analysis

Reader A and B were asked to grade the overall image quality, artifacts, and urinary tract visualization (renal calyces, renal pelvis, ureter on each side as well as bladder) on a 5-point grading scale. The overall image quality was graded as follows: 1, non-diagnostic; 2, major artifact and main duct only partially visible; 3, moderate artifact with blurring or partial lack of duct visualization; 4, slight artifact without loss of diagnostic value; 5, no detectable artifact and excellent duct visualization. The image artifact assessment was divided into two parts: (1) image blur due to respiratory artifacts; and (2) image distortions or local signal change caused by susceptibility artifacts. The two types of artifacts were separately graded as follows: 1, severe artifacts, image not diagnostic; 2, major artifacts, significantly decreased diagnostic ability; 3, moderate artifacts, partially affecting diagnosis; 4, minor artifacts, without affecting diagnosis; 5, no artifacts. The urinary tract visualization was graded as follows: 1, non-visualization; 2, poor visualization with limited diagnostic value; 3, partially visualized; 4, near-complete visualization; 5, excellent clear visualization.

With three readers being blinded to the previous results of qualitative analysis, the two MRU sets (BH-GRASE and RT-FSE) with source and MIP images were reviewed for side-by-side comparison in blind randomized order. Three readers were asked to rank their preference based on diagnostic image quality and confidence: equally prefer both, or prefer set 1, or 2.

### Quantitative image analysis

Two readers (A and C) performed quantitative analysis of the source images of two MRU sequences, with interobserver agreement assessed afterward. To measure the signal intensities (SI), representative slices that depicted the largest area of the dilated renal pelvis, dilated ureter, and bladder were selected, and their SI was measured by applying regions of interest (ROIs) in these slices. ROIs were placed in homogeneous, artifact-free areas of the renal pelvis, ureter, and bladder as well as homogeneous, artifact-free areas adjacent to the pelvis, ureter, and bladder at the same slice, respectively. For the measurement of SIs of the adjacent area, the ROI was placed avoiding other fluid-containing structures. The relative contrast ratio (CR) was selected as a quantitative index to reflect the contrast between the urinary tract (U) and adjacent area (A) [18]. The CR was selected instead of the common CNR calculation based on image noise, as the heterogeneous signal intensity of the background was unreliable and difficult to define [19]. The CR value was calculated using the following formula:

$${\text{CR}} = ({\text{SI}}_{{\text{U}}} - {\text{SI}}_{{\text{A}}} ) \, / \, \left( {{\text{SI}}_{{\text{U}}} + {\text{SI}}_{{\text{A}}} } \right)$$

where SI_U_ and SI_A_ stand for the signal intensities of the urinary tract and adjacent area, respectively. If bilateral dilation of the renal pelvis or ureter was observed, the CR values of both sides were measured and calculated. The final CR values of the renal pelvis or ureter were the mean CR values of both dilated sides.

### Performance evaluation

Two readers (B and C) separately assessed the degree of dilation, obstructive side (unilateral or bilateral), obstructive level (ureteropelvic junction, abdominal ureter, pelvic ureter, intravesical ureter) and defined the imaging features of the obstructive site as benign (smooth, gradual tapered) or malignant (abrupt, irregular cut off) on both MRU protocols. The degree of dilation was defined as follows: mild (pelvis dilation alone), moderate (with mild calyceal dilation), or severe (with severe calyceal dilation) [20].

The reference standard was confirmed by the pathological results of the endoscope biopsy or surgery. For the patients suspicious of benign etiologies whose pathological results were unavailable, a diagnosis of benign obstruction was confirmed by clinical data or laboratory examinations.

### Statistical analysis

All descriptive data are described as the means ± standard deviation (SD). Differences in the acquisition time and CR value between BH-GRASE and RT-FSE were analyzed using paired *t*-test or Wilcoxon signed-rank test after the normality test. A Wilcoxon signed-rank test was used to evaluate the differences in the qualitative scores of overall imaging quality, artifacts, and structure visualization between the two MRU techniques. A two one-sided test of equivalence (TOST) based on the Wilcoxon signed-rank test was performed to assess the equivalence of qualitative scores of the two MRU images, using a ± 0.5 equivalence region [21, 22]. Kappa statistics or intraclass correlation coefficient (ICC) were calculated (1) to measure the interobserver agreement between two readers; and (2) to evaluate the inter-sequence consistency for performance ability between BH-GRASE and RT-FSE. A value of less than 0.20 was considered as disagreement; 0.21–0.40, poor agreement; 0.41–0.60, moderate agreement; 0.61–0.80, good agreement; and over 0.80, excellent agreement. Receiver operating characteristic (ROC) analysis was used to explore the diagnostic performance of the two MRU sequences in differentiating malignant from benign dilation. Sensitivity, specificity, and accuracy were calculated by 2 × 2 contingency tables. Statistical analyses were performed using SPSS software (version 26; IBM) or NCSS software (version 12). *p* < 0.05 was considered to indicate statistical significance.

## Results

### Patients and diagnosis

Among 114 participants, 24 patients were excluded (Fig. 2). Finally, 90 patients (47 men and 43 women; mean age, 49.3 ± 19.1 years; ranges, 18–87 years) were included. The reference standard for the diagnosis of urinary tract pathology was based on subsequent surgery (*n* = 22) and endoscope (*n* = 22). Of these, 21 were diagnosed with malignant lesions and 23 had benign strictures. The remaining 46 patients were clinically diagnosed due to benign considerations based on clinical information, imaging analysis, and laboratory findings (Table 1).

**Table 1:** Demographics of the study population

	n
Population	90
Age (range)	49.3 ± 19.1(18–87)
Sex	
Male	47
Female	43
Cause of dilation	
Malignant (*n* = 21)	
Upper tract urothelial carcinoma	14
Bladder cancer	6
Metastasis	1
Benign (*n* = 69)	
Inflammation	39
Congenital stricture	21
Pelvic lipomatosis	3
Retroperitoneal fibrosis	2
Stone	2
Endometriosis	1
Neurogenic bladder	1

### Acquisition time

The acquisition time was 14.8 s for BH-GRASE and 213.6 ± 52.2 s (range 132–392 s, *p* < 0.001) for RT-FSE. BH-GRASE MRU significantly decreased the acquisition time (93%) within a single breath-hold acquisition.

### Qualitative analysis

Overall, the interobserver agreement was good to excellent (*κ* = 0.701–0.904) between the two readers for the two MRU sequences concerning overall image quality, artifacts, and urinary tract visualization. The detailed kappa values are depicted in Table 2.

**Table 2:** Qualitative analysis scores of the two MRU protocols (means ± standard deviation)

		BH-GRASE	RT-FSE	Kappa	*p*^a^	*p*^b^
Overall image quality	Reader A	4.58 ± 0.72	4.42 ± 0.87	0.712	0.142	< 0.001*
	Reader B	4.48 ± 0.86	4.41 ± 0.91		0.580	< 0.001*
Artifacts						
Respiratory artifact	Reader A	4.70 ± 0.68	4.56 ± 0.82	0.701	0.218	< 0.001*
	Reader B	4.69 ± 0.68	4.51 ± 0.75		0.114	< 0.001*
Susceptibility artifact	Reader A	4.92 ± 0.31	4.98 ± 0.21	0.904	0.025*	< 0.001*
	Reader B	4.93 ± 0.29	4.98 ± 0.21		0.046*	< 0.001*
Structure visualization						
Left calyx	Reader A	4.10 ± 1.46	3.91 ± 1.49	0.770	0.047*	< 0.001*
	Reader B	4.12 ± 1.48	3.97 ± 1.49		0.041*	< 0.001*
Right calyx	Reader A	4.26 ± 1.31	4.03 ± 1.38	0.729	0.028*	< 0.001*
	Reader B	4.18 ± 1.40	3.97 ± 1.42		0.028*	< 0.001*
Left pelvis	Reader A	4.31 ± 1.37	4.29 ± 1.42	0.809	0.870	< 0.001*
	Reader B	4.23 ± 1.44	4.17 ± 1.49		0.399	< 0.001*
Right pelvis	Reader A	4.37 ± 1.32	4.30 ± 1.33	0.749	0.323	< 0.001*
	Reader B	4.27 ± 1.37	4.16 ± 1.41		0.124	< 0.001*
Left ureter	Reader A	3.16 ± 1.63	3.19 ± 1.64	0.775	0.447	< 0.001*
	Reader B	3.00 ± 1.62	3.07 ± 1.57		0.283	< 0.001*
Right ureter	Reader A	3.14 ± 1.57	3.11 ± 1.56	0.766	0.527	< 0.001*
	Reader B	2.93 ± 1.59	2.89 ± 1.53		0.479	< 0.001*
Bladder	Reader A	4.93 ± 0.36	4.92 ± 0.37	0.752	0.317	< 0.001*
	Reader B	4.89 ± 0.46	4.89 ± 0.46		1.000	< 0.001*

*BH-GRASE* breath-hold gradient- and spin-echo, *RT-FSE* respiratory-triggered fast spin-echo

*p value with significance

^a^Difference testing by the Wilcoxon signed-rank test

^b^Equivalence testing by the two one-sided tests (TOST) methods

**Fig. 3:**
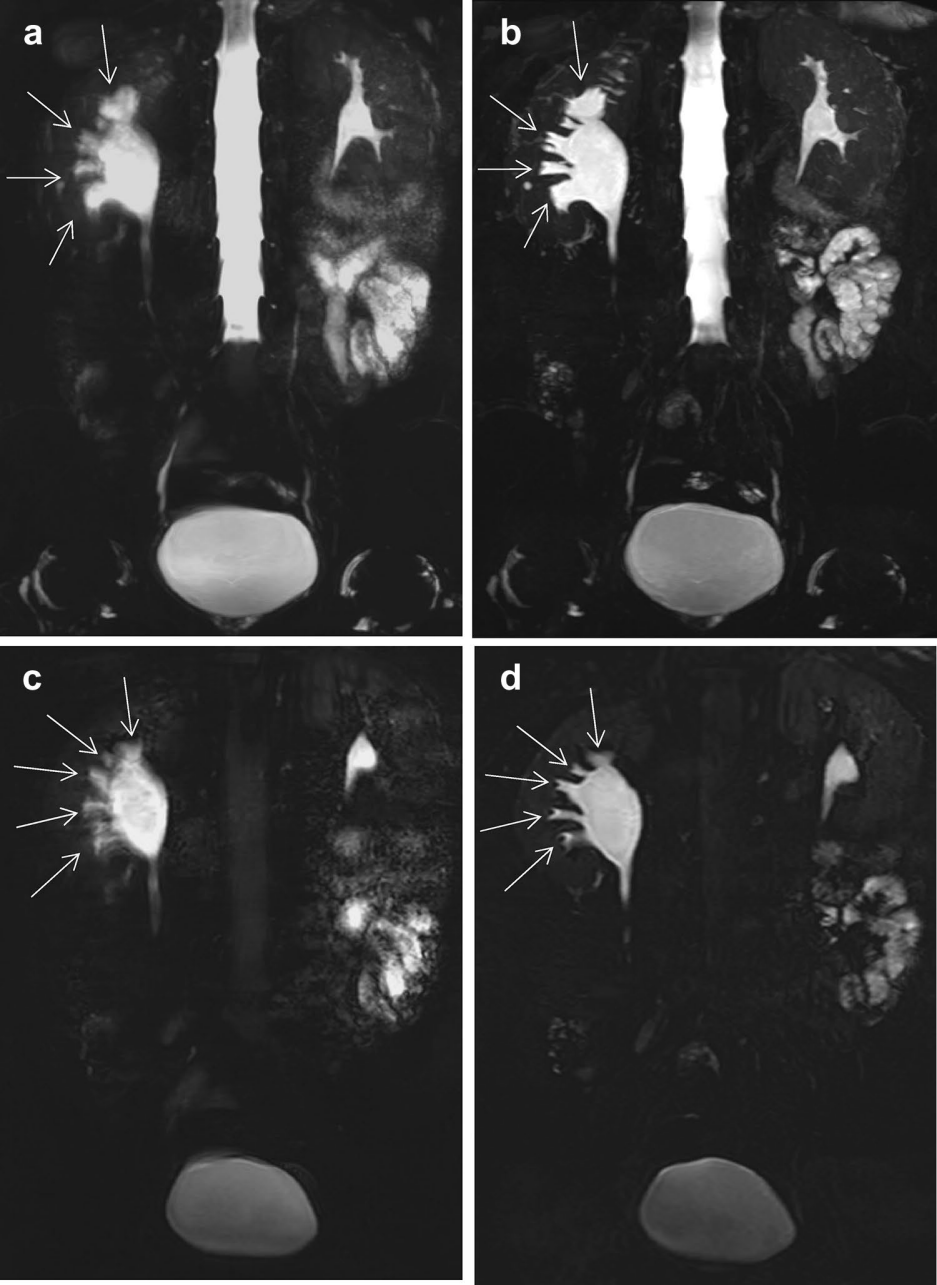
A 49-year-old male with right ureteropelvic junction obstruction. The visualization of dilated right renal calyces and pelvis are blurred due to respiratory artifacts in RT-FSE MRU (**a** MIP; **c** source image), whereas 3D BH-GRASE MRU (**b** MIP; **d** source image) displays them clearly (arrows). The acquisition times were 5 min 8 s for 3D RT-FSE MRU and 14.8 s for 3D BH-GRASE MRU

The qualitative scoring values of image quality, artifacts, and structure visualization for the two MRU protocols are summarized in Table 2. The scores of the BH-GRASE and RT-FSE MRU were found to be statistically equivalent in the qualitative assessment of overall image quality, artifacts (respiratory and susceptibility artifacts), and urinary tract visualization (all *p* values < 0.05). No significant difference was observed between the two MRU sequences in overall image quality, respiratory artifacts (image blur), or visualization of the bilateral renal pelvis, ureter, and bladder (all *p* values > 0.05). For the visualization of bilateral renal calyces, BH-GRASE was better than RT-FSE (*p* < 0.05). On RT-FSE MRU, 10 patients were considered to have moderate to severe image blur (scores ≤ 3) with some decrease of the diagnostic ability, in whom 8 patients had an increased score of 4–5 on BH-GRASE MRU (Fig. 3). The susceptibility artifacts of BH-GRASE MRU were more prone to that of RT-FSE (*p* < 0.05). Five patients showed slight signal loss of the bladder caused by the near gas-filled rectum on BH-GRASE MRU (scores = 4), whereas all of them obtained normal signal intensity at the same location on RT-FSE MRU (scores = 5). One patient with filled renal stones partially affected the observation of the renal calyces and pelvis due to susceptibility artifacts on both MRU images (both scores = 3).

Between two 3D MRU sequences, three readers preferred both protocols equally in most of patients (Reader A: 57/90, 63.3%; Reader B: 60/90, 66.7%; Reader C: 58/90, 64.4%). For the remaining patients, the readers preferred more BH-GRASE (Reader A: 22/90, 24.4%; Reader B: 21/90, 23.3%; Reader C: 23/90, 25.6%) than preferred RT-FSE (Reader A: 11/90, 12.2%; Reader B: 9/90, 10%; Reader C: 9/90, 10%).

### Quantitative analysis

The interobserver agreement was good (ICC_pelvis_ = 0.630; ICC_ureter_ = 0.668; ICC_bladder_ = 0.642). For the quantitative evaluation of urinary tract contrast, CR values were slightly lower on BH-GRASE MRU (CR_pelvis_ = 0.81 ± 0.13; CR_ureter_ = 0.91 ± 0.12; CR_bladder_ = 0.93 ± 0.03) than on RT-FSE MRU (CR_pelvis_ = 0.86 ± 0.09; CR_ureter_ = 0.95 ± 0.04; CR_bladder_ = 0.96 ± 0.02; all *p* values < 0.05).

### Diagnostic performance

**Fig. 4:**
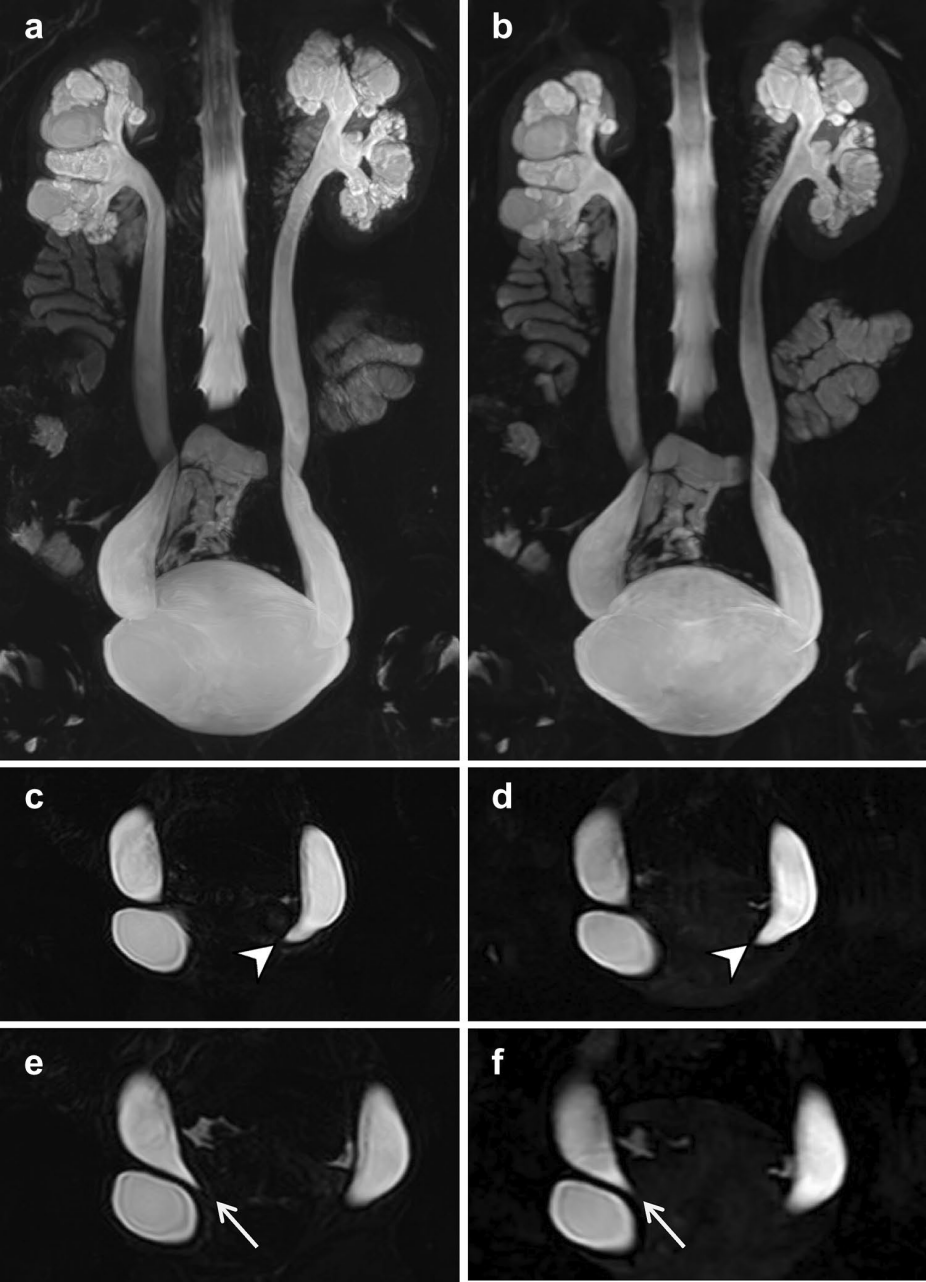
A 27-year-old female with bilateral ureterovesical junction obstruction. Coronal MIP images from 3D RT-FSE (**a**) and BH-GRASE MRU (**b**) allow good visualization of diffuse dilated bilateral collecting systems and ureters. The degree and extent of dilation are clearly exhibited in both MIP images. The oblique coronal MPR images from 3D RT-FSE (**c**, **e**) and BH-GRASE MRU (**d**, **f**) clearly displayed beak-like narrowing at the end of the left ureter (arrowheads) and right ureter (arrows). The acquisition times were 2 min 23 s for 3D RT-FSE MRU and 14.8 s for 3D BH-GRASE MRU

**Fig. 5:**
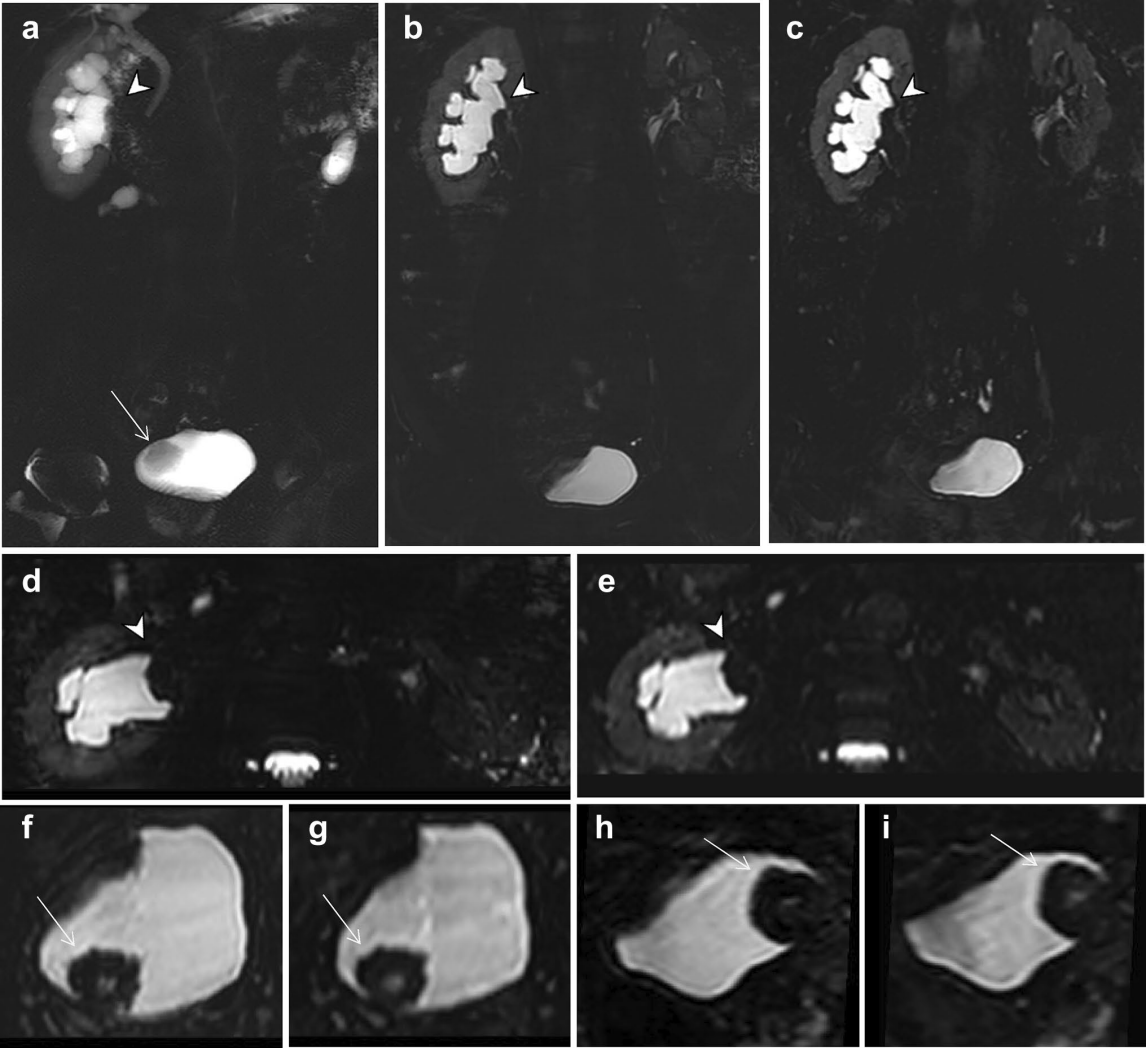
A 68-year-old male with urothelial carcinoma diagnosed by ureteroscopic biopsy. Severe dilation of the right collecting system is observed in source images of 2D thick-slab MRU (**a**), 3D RT-FSE MRU (**b**), and 3D BH-GRASE MRU (**c**). The MPR images from RT-FSE MRU (**d**, **f**, axial; **h**, sagittal) and BH-GRASE MRU (**e**, **g**, axial; **i**, sagittal) equally demonstrate the irregular filling defect in the right pelvis (arrowheads) and bladder (arrows). The acquisition times were 3 min 54 s for 3D RT-FSE MRU and 14.8 s for 3D BH-GRASE MRU

The interobserver agreement was good to perfect (interobserver *κ* = 0.603–1) for the diagnostic performance between the two readers. BH-GRASE MRU achieved a comparable diagnostic capacity with RT-FSE MRU for locating and diagnosing the possible etiology of urinary tract dilation and showed excellent diagnostic consistency (inter-sequence *κ* = 0.924–1) between the two MRU sequences (Table 3). The diagnostic efficiency for the malignant stenosis showed no difference between the two MRU sequences with the AUC of 0.729 (95% CI 0.590–0.867) for reader B and 0.796 (95% CI 0.675–0.918) for reader C. Representative cases are shown in Figs. 3, 4, and 5.

**Table 3:** Diagnostic performance of the two MRU protocols (Reader B/Reader C)

	BH-GRASE	RT-FSE	Kappa	
			Inter-sequence	Inter-observer
Degree of dilation				
Mild	17/13	14/9	0.947/0.924	0.681
Moderate	32/30	35/34		
Severe	41/47	41/47		
Side of dilation			1/1	1
Bilateral	11/11	11/11		
Unilateral	79/79	79/79		
Obstructive level			1/1	0.984
Ureteropelvic junction	44/43	44/43		
Abdominal ureter	14/14	14/14		
Pelvic ureter	15/16	15/16		
Intravesical ureter	17/17	17/17		
Obstructive characteristic			1/1	0.603
Benign	68/64	68/64		
Malignant	22/26	22/26		
Diagnostic efficiency				
AUC (95% CI)	0.729 (0.590–0.867) /0.796 (0.675–0.918)	0.729 (0.590–0.867) /0.796 (0.675–0.918)	–	–
Sensitivity (%)	85.7/84.3	85.7/84.3	–	–
Specificity (%)	60/75	60/75	–	–
Accuracy (%)	80/82.2	80/82.2	–	–

*BH-GRASE* breath-hold gradient- and spin-echo, *RT-FSE* respiratory-triggered fast spin-echo, *AUC* area under the curve, *CI* confidence interval

## Discussion

As far as we know, our study is the first investigation of 3D GRASE MRU. Conventionally, 3D RT-FSE MRU is time-consuming, and the image quality is prone to be affected by variable respirations. In this study, the performance of two different MRU methods, based on breath-hold GRASE and respiratory triggered, respectively, was compared. 3D MRU was successfully performed within a single breath-hold (14.8 s) using the GRASE technique. Compared with RT-FSE MRU, BH-GRASE MRU showed comparable image quality and improved urinary tract visualization by reducing motion-related image blur. Additionally, BH-GRASE MRU allows successful determination of the level of urinary tract obstruction, the degree of dilation, and the possible cause of the obstruction, which shows excellent agreement to conventional RT-FSE MRU.

The thin-slice 3D images, compared with 2D MRU, allow reconstruction into any imaging plane at the expense of acquisition time but may provide a more detailed assessment of anatomic structures [23] (Fig. 5). The GRASE sequence, proposed in the early 1990s [11], is a combination of spin-echo and gradient-echo sequence. It provides a shorter acquisition time than conventional FSE, due to the interleaving of the EPI readout between two consecutive 180 pulses (Fig. 1). GRASE is less prone to field inhomogeneity and less demanding for gradient performance than EPI [13]. In this study, we accelerated the acquisition of 3D MRU by adopting an FSE factor of 35, an EPI factor of 7, and a SENSE factor of 3. Acceleration of the 3D MRU technique not only increases patient tolerance but also decreases motion-related artifacts.

Based on our qualitative evaluation, there were no significant differences between the two MRU protocols in image quality and urinary tract display including the renal pelvis, ureter, and bladder. The qualitative scores on RT-FSE were inferior to those of BH-GRASE for the observation of renal calyces. The blur artifacts caused by the respiratory movement have a negative impact on the display of renal calyces, which was likely due to the renal calyces being more adjacent to the diaphragm. Although we trained the breath for every patient before the examination, a small number of patients were unable to cooperate with the breath instructions well during the scan resulting in the respiratory-related image blur.

The image quality, artifacts, and urinary tract visualization showed statistical equivalence by the two one-sided tests. Based on the results, BH-GRASE MRU could provide comparable image quality with conventional RT-FSE MRU. Meanwhile, both 3D MRU protocols could be equally preferred for most of the cases (63–67%), considering the subjective preferences of three readers. For the rest of the patients, the readers preferred either BH-GRASE MRU (23–26%) or RT-FSE MRU (10–12%). In summary, the BH-GRASE MRU could be used as a clinically feasible 3D MRU protocol for nearly 90% of patients and showed equivalent or better performance than the conventional RT-FSE MRU, whereas a small percentage of patients performed better on RT-FSE MRU, possibly due to the poor cooperation of the breath-hold.

Despite respiratory-related artifacts, the GRASE sequence with the composition of gradient-echo may be more sensitive to susceptibility artifacts of metallic foreign bodies such as surgical clips than the FSE sequence. In this study, no susceptibility artifact from the metal was observed on two sets of MRU images, as we only included patients without any history of surgery. However, we found that the gas in the bowel or renal calcified stones may produce some susceptibility artifacts, which was a little more prominent in GRASE, but without decreasing the diagnostic ability compared with the FSE sequence. Although we did not include patients after surgery, the surgical clips used in the urinary tract are usually made of titanium, which is nonmagnetic and does not cause obvious susceptibility artifacts. Further studies are needed to assess the performance of GRASE MRU in patients with surgical implants for postoperative evaluation.

For the diagnostic performance of urinary tract dilation, there was no difference between the two MRU protocols except for the judgement of the degree of dilation. The number of mild and moderate hydronephrosis was slightly different between the two MRU protocols. The standard to differentiate mild or moderate degree is whether the renal calyces are dilated. The degree of a few patients was diagnosed mild in BH-GRASE MRU, but moderate in FSE MRU. These patients had respiratory-related artifacts and poor visualization of renal calyces on RT-FSE MRU, while image quality improved on BH-GRASE MRU. The dilated degree of renal calyces may be potentially misjudged by blurring artifacts. However, there was no difference in the diagnostic performance of the obstructive level and image features between the two protocols as the pathological site of urinary tract obstruction was usually at the ureter, which was less prone to respiratory-related artifacts. There was no difference in the diagnostic efficiency for differentiating malignant stenosis from the benign ones between the two MRU sequences. However, it should be noted that the static-fluid MRU sequence usually requires other MRI sequences to make a final diagnosis. Additionally, we did not obtain the final pathological diagnosis of every patient. Therefore, the diagnostic efficiency of the MRU protocol needs further evaluation.

In addition to artifacts, the spatial resolution and contrast may also affect the image quality and diagnostic ability. In our study, 3D BH-GRASE had slightly larger voxel size and thicker slice thickness than RT-FSE. There is a balance between spatial resolution and acquisition time. The spatial resolution was somewhat sacrificed due to the limitation of one breath-hold time. Additionally, BH-GRASE with shorter TR and TE will lead to lower T2 contrast which was consistent with the lower CR values of BH-GRASE in our quantitative analysis. However, the slightly decreased spatial resolution and lower contrast of BH-GRASE MRU did not affect qualitative scores and diagnostic performance. The clinical application of BH-GRASE may not be inferior to that of RT-FSE.

As the multi-parametric MR urography acquisition requires coverage of the entire urinary tract from the abdomen to pelvis with several types of sequences [24], it is usually time-consuming with an acquisition time of approximately 30 to 40 min. As the 3D BH-GRASE MRU is quick and easy to perform, adding it into the routine multi-parametric MR urography protocol may provide not only a broad overview of the urinary tract but also more diagnostic information without increasing the time burden. We think the results of our study may provide a clinically feasible option for 3D MRU sequences. As BH-GRASE MRU increased the scan efficiency from the previous 2–6 min to 14.8 s, we preferred the 3D BH-GRASE MRU sequence as the first selection of the 3D MRU protocol.

There are several limitations to our study. First, there was a selection bias because we only included patients with dilation of the urinary tract for evaluation, as the clinical application of MRU is less beneficial in patients with non-distended urinary tract [25]. Second, we did not compare GRASE with other single breath-hold techniques, such as thin-section 2D single-shot FSE sequences or breath-hold compressed sensing (CS) sequences. Third, the acquisition parameters were partly different between the two MRU protocols due to optimization for single breath-hold acquisition. Fourth, our study included patients regardless of their ability to hold their breath. We did not evaluate the patients’ respiratory ability before the examination. The image quality of the breath-hold sequence may be decreased if the patients can not cooperate to hold their breath. For these patients, whether we repeat the breath-hold MRU after training again or change it to RT-FSE MRU needs more investigations. Future studies are needed to optimize the MRU protocols and parameters for various groups of patients.

## Conclusion

In conclusion, our study demonstrates the clinical feasibility of BH-GRASE in 3D static-fluid MRU scanning for depicting urinary tract obstruction and dilation, as it significantly shortens acquisition time to a breath-hold without sacrificing image quality. The BH-GRASE sequence may be preferred as a clinically feasible 3D MRU protocol.
